# Design, Fabrication and Analysis of a Hybrid-Order Monolithic Imaging Diffractive Lens on a Germanium Substrate

**DOI:** 10.3390/mi14030657

**Published:** 2023-03-15

**Authors:** Yidi Zheng, Boping Lei, Bin Fan, Junfeng Du, Jiang Bian, Lihua Wang, Yuchen Liu, Shanghong Guan, Dun Liu, Qian Luo, Hu Yang, Hao Zhang, Chao Hu

**Affiliations:** 1 Institute of Optics and Electronics, Chinese Academy of Sciences, Chengdu 610200, China; 2 University of Chinese Academy of Sciences, Beijing 100049, China

**Keywords:** diffractive optical elements, chromatic aberration, infrared optical system, single-lens imaging, NETD

## Abstract

Diffractive optical elements are gradually replacing some conventional optical elements and becoming a key component of optical systems due to their unique phase modulation function. However, the imaging performance will be reduced due to the fact that this single-sided microstructured lens still produces chromatic aberration. Therefore, the key issue for the application of diffractive optical elements in optical systems is the reduction of chromatic aberration, and diffractive lenses with double-sided microstructures are proposed as a solution. This research describes the design and analysis of a 70-mm-diameter, 296-mm-focal-length double-sided microstructured hybrid-order monolithic imaging diffractive lens operating in the mid-wave infrared region (3.7–4.7 μm). The design minimizes chromatic aberration by up to 30 times compared to a standard harmonic diffractive lens and improves the image performance of a single-lens optical system operating in the infrared range. Experiments indicate that this design is capable of achieving single-lens imaging with high sensitivity for optical systems with a measured NETD ≤ 50 mK. The analysis of the experiments yielded suggestions for future research.

## 1. Introduction

Because of their excellent properties of thinness and high design freedom, diffractive optical elements (DOEs) are key devices in optical systems for achieving lightweight optical systems. DOEs produce diffraction effects by varying the phase. Researchers have realized a variety of applications, including compound eye optical systems [1], beam shaping [2,3,4], and spectral confocal systems [5]. However, chromatic aberration is introduced into the imaging process in optical imaging systems due to the strong negative dispersion properties, which reduces the imaging performance of DOE in wide band optical imaging systems and is limited in wide band optical imaging system applications.

To solve the chromatic aberration phenomenon induced by DOE into optical systems, multilayer DOE methods, such as double-layer DOE and triple-layer DOE, were developed to improve image performance. Multiple phase modulation enhances the diffraction efficiency of multilayer DOE. To increase the optical system’s bandwidth by solving the issue of degraded diffraction efficiency when the DOE operational wavelength deviates from the design wavelength, Yoel Arieli et al. originally suggested the concept of chromatic aberration reduction in broad band utilizing double-layer DOE [6], which uses two DOEs made from different materials to reduce chromatic aberration. Qingfeng Cui et al. analyzed the relationship between the integrated multicolor integral diffraction efficiency (PIDE) and the design wavelength pairs of multilayer DOEs [7] and combined the multilayer DOEs and the maximum integrated PIDE to determine the design wavelengths and required materials for improved design outcomes. Xie H. et al. [8]. constructed and analyzed a triple-layer DOE made from carefully selected optical materials to replace the air gap of a double-layer DOE. However, the system’s structure is complicated, and its manufacture is expensive.

Harmonic diffractive optical elements (HDOEs), obtained by modifying the phase depth factor M, are essential for optical imaging systems in order to improve imaging performance. HDOEs contain fewer loop bands and greater microstructure depth, which allows it to generate harmonic lengths and increase imaging performance. HDOEs are frequently utilized and have become a popular area of study. Sweeny et al. [9]. initially proposed the HDOE idea and fabricated a lens that approaches the diffraction limit for ten discrete visible light wavelengths. Recent research combining HDOE with image processing techniques [10,11,12,13] has demonstrated imaging performance comparable to that of traditional optical systems. However, these solutions do not take into account the application situation of large-aperture optical systems, in which large-aperture primary mirrors with strong achromatic performance can solve the problem of optical system lightweighting in the field of telescopic optical systems.

**Figure 1:**
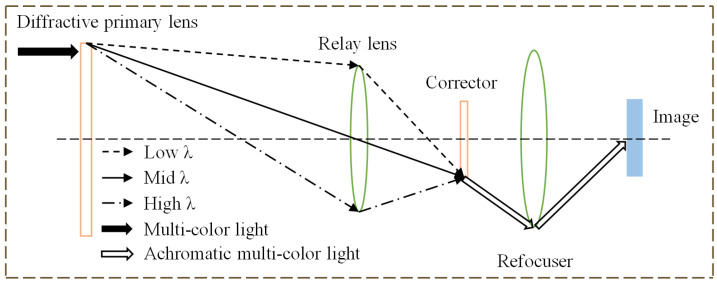
Schematic diagram of Schupmann achromatic structure.

As demonstrated in Figure 1, the diffraction achromatic structure based on the Schupmann structure is the current conventional solution for telescopic optical systems. The diffraction achromatic structure based on the Schupmann structure as the current classical solution in the area of telescopic optical systems, as shown in Figure 1, can effectively eliminate the chromatic aberration problem and enhance image performance close to the diffraction limit. Researchers have conducted extensive studies using various primary diffractive lens combined with Schupmann achromatic structures [14,15,16,17]. Chuanwang He et al. [18] adapted the Schupmann achromatic structure to an off-axis reflection system to achieve a loose tolerance and got outstanding imaging performance in the 612.8–652.8 nm region approaching the diffraction limit. The complicated arrangement of follow-up mirrors and long optical path in the achromatic structure contribute to the heavy weight of the optical system based on the Schupmann structure, making it challenging to achieve lightweight optical systems for telescopic applications.

Zichan Wang et al. proposed using MODE in the astronomical R-band (589–727 nm) as the primary mirror design, and custom materials were used to improve the achromatic performance of the primary and subsequent mirror sets, reducing longitudinal chromatic aberration in combination with subsequent achromatic structures while maintaining the effective focal length and numerical aperture of the system [19,20].

A hybrid-order monolithic imaging diffractive (HMID) lens design method working in the mid-wave infrared band is suggested in this study to overcome the chromatic aberration problem by introducing a diffractive lens into the optical system and enabling single-lens imaging. HMID is created by partition optimization and contains a double-sided microstructure. In the lens-partitioning optimization procedure, damped least squares is employed to ensure that each region’s achromatic performance is optimal. The higher-order diffractive plane and the first-order diffractive plane are designed on the front and rear sides of the HMID, respectively, and the HMID is capable of multiple phase modulation. This two-sided microstructure architecture increases the infrared band achromatic capacity while reducing the imaging system’s complexity. The experimental results reveal that the HMID is capable of single-lens imaging and serves as a benchmark for monolithic infrared imaging optical systems. Simulation and experimental results show that the proposed strategy has significant advantages in achromatization.

## 2. Principles and Design

### 2.1. Principle of High-Order Harmonic Diffraction

HDOE is a generic variant of DOE, the difference between the two is the phase depth factor: HDOE is M (M is an integer higher than 1), whereas normal DOE is 1 with a first-order diffraction surface (FODS). For greater M, the HODE surface is a high order harmonic diffraction surface (HODS) with increased microstructure and decreased number of zones. The DOE has theoretical 100% diffraction efficiency only at the design wavelength, but the HDOE can achieve theoretical 100% diffraction efficiency at both the design wavelength and multiple harmonic wavelengths, enabling HDOE to perform better in broad band imaging optical systems.

**Figure 2:**
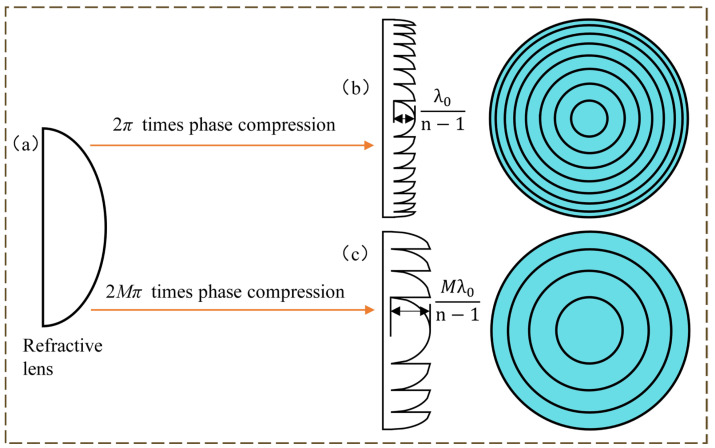
(**a**) Ideal refractive lens; (**b**) Schematic diagram of DOE; (**c**) Schematic diagram of HDOE.

The difference between the two is shown in Figure 2, conventional lenses get the phase of DOE and HDOE after phase compression, and the phase compression equation is:

(1) $$\varphi_{doe}=mod_{2\pi M}\left(\varphi_{lens}\right)$$

where $\varphi_{doe}$ is the compressed phase, $\varphi_{lens}$ is the continuous phase of the refractive lens, *mod* is the remainder operation with $\varphi_{lens}$ as the divisor, and M × 2π is the divisor.

The vector height of the microstructure can be obtained by relating the phase to the optical range as follows:

(2) $$\varphi_{lens}=\left(n-1\right)\times d\times \frac{2\pi }{\lambda }$$

where *n* is the index of refraction of the lens material at the operating wavelength and d is the vector height of the microstructure of the diffractive optical element.

For a system that images objects in the visible spectrum (400–700 nm) with a center wavelength of 550 nm, a DOE with *M* = 1 exhibits > 50% Δ*f* change in focus [19]. For the optical system utilized in this study to image objects in the mid-infrared region (3–5 μm), the DOE displays around 79% Δ*f* fluctuation in focus. The chromatic aberration induced by the fluctuation in focus emerges in the IR picture as a dense haze and blur, which is unacceptable for an imaging system. Whereas HDOE with HODS displays reduced focal dispersion, a system with *M* = 160 in the same operating band exhibits barely 0.6% Δ*f* focal variation over the whole band, with multiple harmonic lengths $\lambda_{p}$ being focused to $f_{0}$, according to Equation (3):

(3) $$\lambda_{p}=M\lambda_{0}/p$$

where *p* is an integer that specifies the diffracted order.

In this research, HODS was utilized to achieve smaller focus fluctuations, which led to a significant reduction in the type 2 longitudinal chromatic aberration (LCA) of the optical system, hence enhancing imaging performance, and bigger M, which led to fewer zones and easier optimization. Additional factors impacting optical system imaging performance, such as spherical aberration and, as a result, spherochromatic aberration, are also considered; however, because aberrations are more difficult to rectify in single-lens imaging systems, only chromatic aberration is discussed in this study.

### 2.2. Design Method

First of all, it is necessary to find out what kind of chromatic aberration there are in order to achieve the purpose of eliminating it. Different wavelengths correspond to different focal lengths, which is known as focal dispersion or LCA, is the primary sources in diffractive optical systems. The LCA of a diffractive optical system is composed of two parts: a type 1 LCA created by the dispersive qualities of the glass and FODS, and a type 2 LCA formed by the diffraction properties present on the HODS surface, as explained in Section 2.1.

Since the goal of this study is to finish the single-lens design and experiment of HMID and the type 2 LCA is less than the type 1 LCA over the whole spectral range, only the type 1 LCA elimination is considered. To eliminate type 1 LCA, a HODS with *M* = 160 was utilized on the front surface and a FODS was employed on the rear surface.

Using HODS as a front surface not only provides single-wavelength co-phase at the design wavelength $\lambda_{0}$ = 4.2 μm, but also introduces more harmonic wavelengths into the optical system in order to achieve reduced focus fluctuations. The classical notion of achromatic aberration is obtained for each HMID region, which can be interpreted as a refractive/diffractive hybrid achromatic structure that eliminates material and FODS chromatic aberration. In this study, germanium is employed because it has a high transmittance in this band and readily passes energy detection criteria. It is easier to produce than silicon, a brittle material, and less expensive than zinc selenide and zinc sulfide. It also has the advantage of being simple to process.

**Figure 3:**
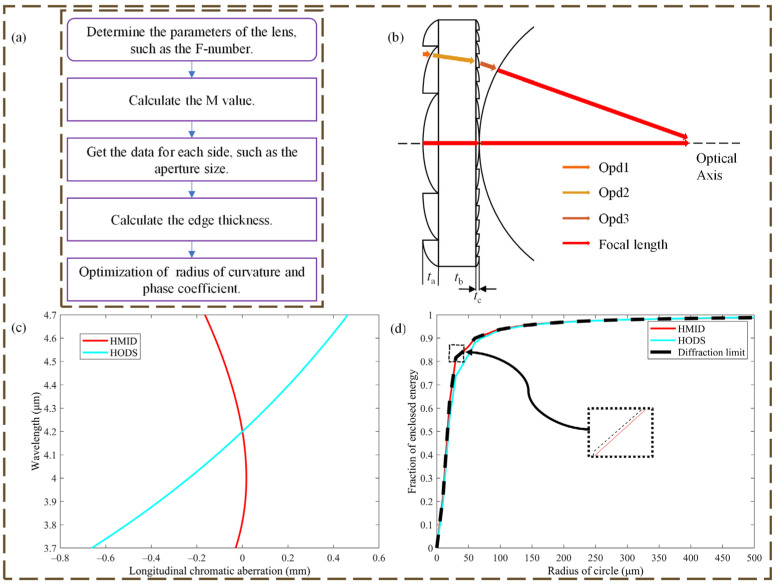
(**a**) Design flow chart of HMID; (**b**) Layout of HMID includes HODS with thickness *t*_a_ = 0.225 mm and a *t*_b_ = 4.714 mm germanium substrate with a *t*_c_ = 1.39 μm FODS on the back surface. A schematic diagram of the calculation of ray tracing is included; (**c**) LCA comparison of HMID and HODS, where the maximum reduction is 30 times; (**d**) Comparison of encircled energy.

The HMID is a 69 mm diameter aperture, f/4.21 three-zone lens with a 296 mm focal length and is designed for evaluation with an object at infinity, as shown in Table 1, where FODS is designed on the second surface. The optical system and HMID parameters are shown in Table 1 and Table 2, respectively. The diameter was designed using the processor’s material data, and the focal length was calculated using the F-number, which was chosen because our existing IR-cooled detectors operate in the 3.7 to 4.7 μm band. The HMID lens layout is shown in Figure 3a. The HMID is rotationally symmetric, and the whole face shape may be determined by executing a rotationally symmetric operation on the cross-section shown in Figure 3b. We also show a ray-tracing model used to solve for the light range at different points on the HMID surface in this figure. The use of this model will be described in Section 2.3.

The dispersion performance comparison and the encircled energy comparison of HMID and HOSD are shown in Figure 3, these dispersion measurements are evaluated in zone 1 because zone 1 concentrates more energy than the other two zones combined, which is essential for infrared optical systems. In Figure 3b, a comparison of the dispersion performance before and after lens optimization indicates that the dispersion performance of HMID is lowered by a factor of up to 30 times to HODS, and the encircled energy plot in Figure 3c is nearly perfect for HMID. This research focuses on the dispersion performance and encircling energy of the on-axis field of vision due to the fact that the system is designed to image objects at infinity. This is excellent for imaging with a single lens in infrared optical systems. Because this achromatic technique is based on the features of HDOS on FODS rather than on this system, it can be applied to other systems with diffractive lenses.

The nominal value of NETD of the infrared-cooled detector we used is used as a reference, and the NETD of the ideal infrared optical system is ≤25 mK. However, energy loss in the actual scenario will cause the actual NETD to differ from the ideal, so the desired NETD of the HMID design proposed in this paper is ≤35 mK. 

### 2.3. Sensitivity Analysis

The design proposed in this study is machined using the diamond turning method with germanium material as the substrate. Previous research has demonstrated that the machining cannot produce a physical object that is as perfect as the design [21], so a tolerance analysis or sensitivity analysis is required prior to machining. In this section, a method for analyzing the sensitivity of HMID based on ray tracing is suggested, which ultimately serves as a reference for the real machining.

The light propagation process is divided into three segments, as shown in Figure 3b), the parallel light incident at infinity gets the first optical path difference (Opd1) from the nearest equiphasic plane to the incidence point of the lens, after refracting at the incidence point and entering the lens and propagating to the next plane to get Opd2, after refracting through the second plane and propagating to the nearest equiphasic plane in the air to get Opd3. Opd are the optical path difference disparities between the sought-after light and the highest point of the vector height in the HODS area. We compute and evaluate the peak and valley (PV) values of the light range difference for each division at the design wavelength $\lambda_{0}$, which should ideally be equal to zero. We relax the PV value to $\lambda_{0}/4$ to calculate the new zone vector height or width based on the actual processing situation. The difference between the zone data under the theoretical PV value and the zone data after relaxing the PV value is the maximum vector height or width that HODS can tolerate during the processing error. The same method is applied to the sensitivity analysis of the thickness of the HMID substrate, and the results indicate that the substrate thickness has a negligible impact on the HMID optical path, and a thickness change of at least 1.5 mm is required for the PV value of the optical path difference in a single region of the HMID to become $\lambda_{0}/4$. The sensitivity value of the substrate thickness is not included in Table 1 since it is similarly extremely tiny in the case of processing.

**Figure 4:**
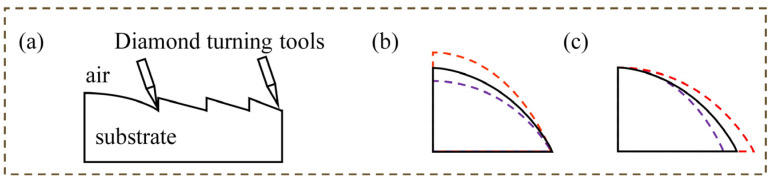
(**a**) Schematic diagram of diamond turning process; (**b**) Possible errors in thickness during processing, with the red dashed line representing a potential higher height change and the purple dashed line representing a potential lower height change; (**c**) Possible errors in thickness during processing, with the red dashed line representing a potential wider height change and the purple dashed line representing a potential narrower height change.

Table 3 provides the exact data for the four causes and errors that result in the shift of PV values to $\lambda_{0}/4$, as seen in Figure 4b,c.

Through the results of sensitivity analysis, HMID’s tolerance for specific machining errors is updated, and these findings serve as an essential reference for the actual machining process.

## 3. Simulation Analysis of the Error

We processed the HMID after design and analysis, and we also examined the errors that may have affected the imaging performance of the HMID during the experiment. Material error, detector error, and processing error were all considered. Material error refers to the inconsistency of refractive index inside the optical material as a result of its inhomogeneity. However, material error has less of an impact on HMID due to the thin material and the greater tolerance of the infrared optical system to material error. Detector error refers to the repeatability error of the detector itself. However, the error is negligible and does not impact HMID imaging in this study. Processing error relates to the fact that real-world processing and design typically differ, as described in Section 2. The phase modulation performance of the HMID may be impacted by the imperfect surface shape of the lens acquired after processing, which in turn impacts the imaging performance. Therefore, processing mistakes are likely to impact the real imaging results and subsequent parameter measurements, so it is essential to test and assess the HMID’s actual surface form.

**Figure 5:**
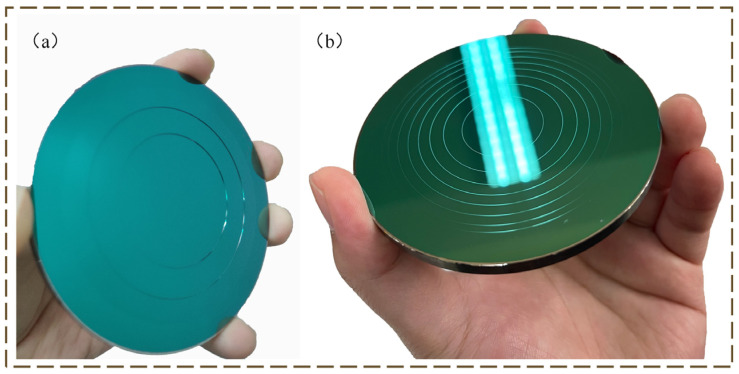
(**a**) A physical view of the front of the HMID; (**b**) The physical picture on the back of HMID.

**Figure 6:**
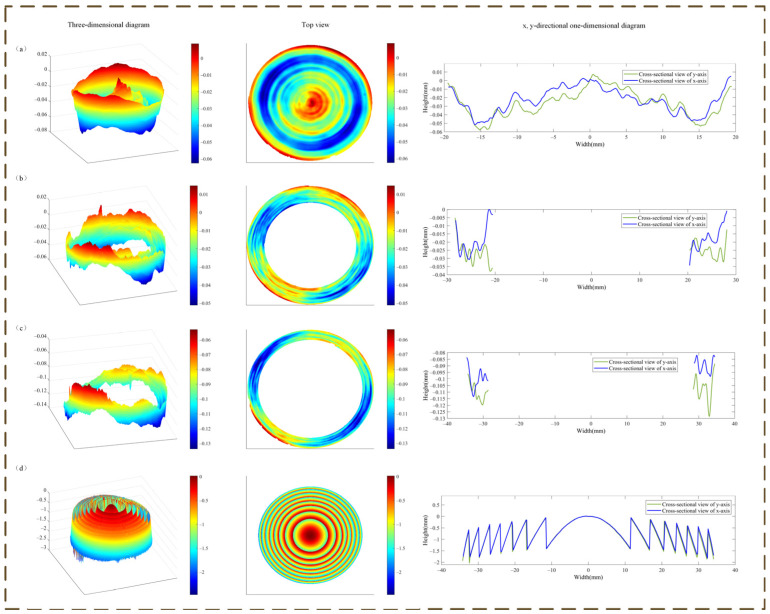
Test results of HMID lens. (**a**) Test results for the first zone of HODS; (**b**) Test results for the second zone of HODS; (**c**) Test results for the third zone of HODS; (**d**) Test results for the FODS.

The completed and coated HMID specimen is shown in Figure 5. The surface profile of the HMID was measured using LuphoScan, and the results are depicted in Figure 6. The three parts of the HODS are measured independently, while the entire FODS is measured to determine the phase coefficients. The procedure results in a disparity between the measured physical surface profile and the designed surface profile. On the basis of the data displayed in Figure 6, we anticipated the probable reasons for mistakes in the machining process. The selection of diamond tools and the setting of parameters such as the quantity of feed during turning were examined as possible sources of machining errors.

The actual FODS measurements are given in Figure 6d, and the measured data are extracted to compare with the design values, which are computed using the phase coefficient, Equation (4):

(4) $$\Phi =M\sum_{i=1}^{N}A_{i}\rho^{2i}$$

where *M* is the diffraction level, *A* is the phase coefficient, *ρ* is the normalized radius, and the definition of *ρ* is given by Equation (5):

(5) $$\rho =\frac{r}{R}$$

where *r* is the radius of each zone and *R* is the maximum radius of the diffractive optical element.

**Figure 7:**
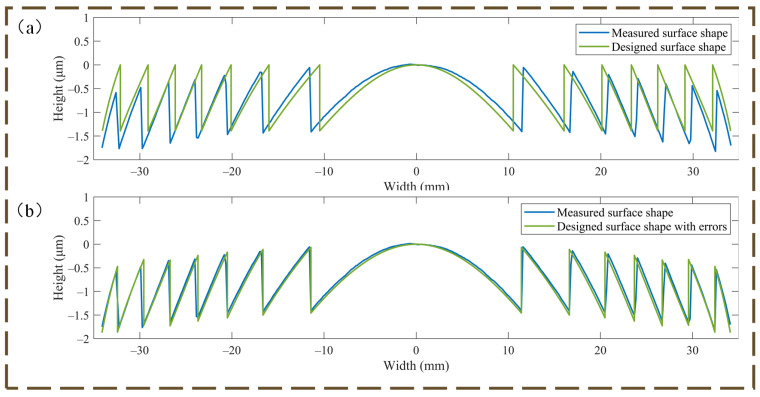
(**a**) Comparison of the measured and designed surface shapes of FODS; (**b**) Comparing the actual measured surface shape and combining potential errors, the data are more consistent.

The theoretical and practical comparison is shown in Figure 7a.

Errors were present in both the zone’s height and breadth. Due to the real processing, the width error dropped progressively from the inside to the exterior, but the height error grew gradually. The highest and average width error values are 0.927 mm and 0.5229 mm, while the greatest and average height error values are 0.7652 μm and 0.3533 μm, respectively.

To reproduce the effect of the error on the HMID, we read out the actual machining radius of each zone and then back-propagated the actual phase coefficients using Equation (4), replacing the design value with the phase coefficient, and the results of the comparison with the design value after adding the width error are shown in Figure 7b, where the maximum and average values of the difference between the measured face shape in the zone become 0.168 mm and 0.052 mm after adding the zone width error. The results are decreased by almost 90% from the measured error average, demonstrating the efficiency of the aforementioned procedure, and this discrepancy may be ignored because HMID operates in the infrared band.

The actual procedure introduces height error into the FODS, as shown in Figure 7a, with maximum and average height error values of 0.7652 μm and 0.3533 μm, respectively. As a result of the rising HMID height inaccuracy from inside to outside, a “concave lens” is placed between the microstructure and the substrate to replicate the real situation. In actuality, the radius of curvature of the “concave lens” is predicted to be 887,582.2 mm, hence its center thickness is negligible. The comparison with the design value after accounting for the height error is given in Figure 7b, where the highest and average values of the difference between the zone and the measured surface shape are 0.118975 μm and 0.0512 μm, respectively. The result is around 85.5% smaller than the average of the measured errors, demonstrating the method’s validity, and this disparity may be neglected because HMID operates in the infrared spectrum.

**Figure 8:**
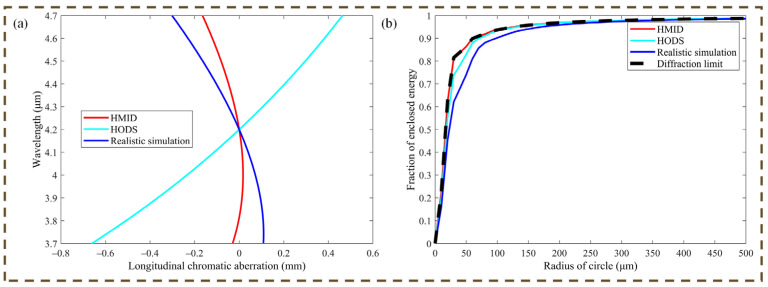
Realistic simulation parameters of HMID taking measurement inaccuracy into consideration. (**a**) LCA comparison against the design value, chromatic aberration is still in the small range; (**b**) Enclosed energy comparison against the design value.

The HODS differs from the design, and the aspheric formula does not accurately depict its surface shape, thus the 36-term Zernike coefficient fitted with MetroPro 8.3.5 software was utilized to express the HODS acquired through diamond turning. Comparison of enclosed energy of zone 1 is shown in Figure 8, the maximum drop in the simulated optical system with inaccuracy is about 23.6% from the design value. Larger LCA is introduced into the optical system due to the presence of machining errors, but the realistic simulated LCA shown in Figure 8a is still smaller than the LCA before color correction. This is still acceptable for the optical system in this paper.

This section analyzes the machining of the HMID in two parts, and the simulation results in Section 2.2 demonstrate that the imaging performance of the HMID in this paper of the design value is good. However, according to the parameters of the optical system after introducing errors in this section, the imaging performance of the physical object may be degraded due to the presence of machining errors, but still maintains a small LCA and good performance of the enclosed energy. The imaging experiments of the HMID will be illustrated in Section 4.

## 4. Experiment

In Section 2, an HMID is developed for use in an infrared imaging system, and this HMID has been manufactured using the diamond turning process on a germanium material substrate. In this Section, the capability of the HMID to conduct single-lens imaging and its imaging performance will be evaluated. As a proof-of-concept experiment, the noise-equivalent temperature difference (NETD) was introduced to test the performance of HMID in the infrared band; the result represents the smallest temperature difference that can be detected by HMID. NETD is a crucial criterion for infrared optical imaging systems. The calculation method and results of NETD will be presented in this Section.

NETD refers to the temperature difference between the target area and the background area of the experimental target. The ratio of the output signal at the center of the field of view to the root mean square noise of both is one when the infrared thermal imaging system is observing a specific image target. According to the definition, the NETD testing equation can be written as Equation (6):

(6) $$NETD=\frac{\sigma }{SiTF}$$

where σ is the root mean square noise; SiTF is the signal transfer function.

The NETD test procedure is as follows, according to Equation (6): the temperature difference signal under different temperature differences is created by managing the temperature change of the blackbody, and the corresponding relationship function SiTF is obtained; the root mean square noise is detected in the signal region or the background region; and the final NETD measurement result is calculated from Equation (6).

SiTF is one of the most essential technical indicators for evaluating the infrared imaging optical system; the response rate function of the infrared imaging optical system is determined when the target size is fixed and the target intensity is variable, SiTF is the slope obtained by fitting the linear region of the function, we use the least squares method for fitting in this paper, as in Equation (7):

(7) $$SiTF=\frac{N\sum_{i=1}^{N}\Delta V_{i}\Delta T_{i}-\sum_{i=1}^{N}\Delta V_{i}\sum_{i=1}^{N}\Delta T_{i}}{N\sum_{i=1}^{N}{\left(\Delta T_{i}\right)}^{2}-{\left(N\sum_{i=1}^{N}\Delta T_{i}\right)}^{2}}$$

where *N* is the quantity of test data; $\Delta V_{i}$ is the output voltage of the infrared imaging optical system, and $\Delta T_{i}$ is the target-background temperature difference.

**Figure 9:**
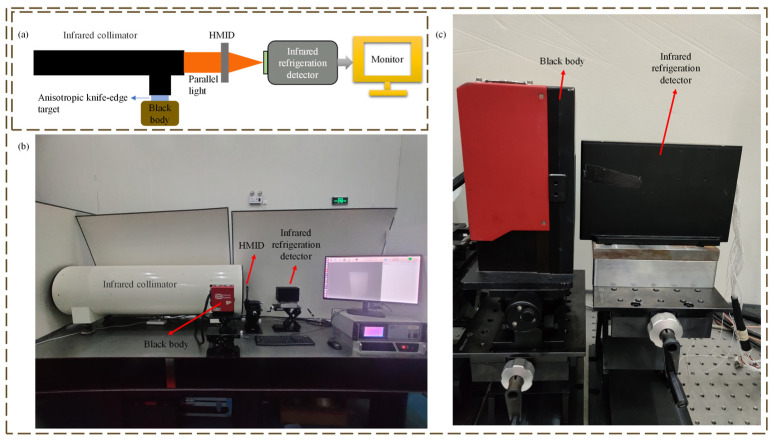
(**a**) Schematic diagram of the optical system used to measure the NETD parameters of HMID; (**b**) Actual optical system diagram for NETD parameter measurement of HMID; (**c**) Actual optical system diagram for measuring the detector’s NETD.

Figure 9 captures the actual optical system diagram for measuring the NETD of detector and HMID. The NETD was computed using earlier research [22,23,24,25] and is not detailed in this paper. The photographs used to compute the NETD were taken at room temperature of 22 °C at intervals of 0.5 °C, and 100 photographs were acquired sequentially at each temperature, using an area of 50 × 50 pixel points per image for the computation; the final measured values are provided in Table 4.

**Figure 10:**
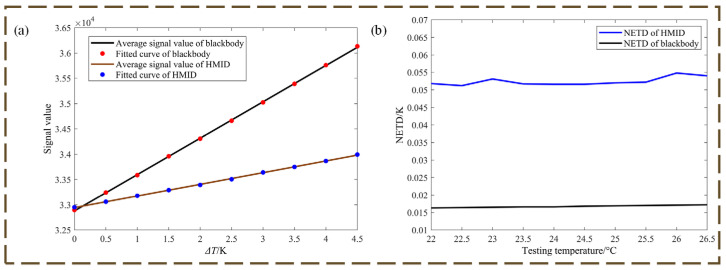
(**a**) Curve chart of system SiTF; (**b**) Curve chart of system NETD, the data are flat.

Figure 10 exhibits the optical system’s SiTF and NETD plots. The average of the measured NETD values is 16.74 mK for the infrared-cooled detector we used; the nominal NETD of the detector is less than 16 mK. The measured NETD value of HMID at around 50 mK is approximately three times the nominal NETD value, and the performance is comparable to certain uncooled detectors. While there is a difference with the ideal circumstance, it is still more sensitive and demonstrates the right design of HMID.

**Figure 11:**
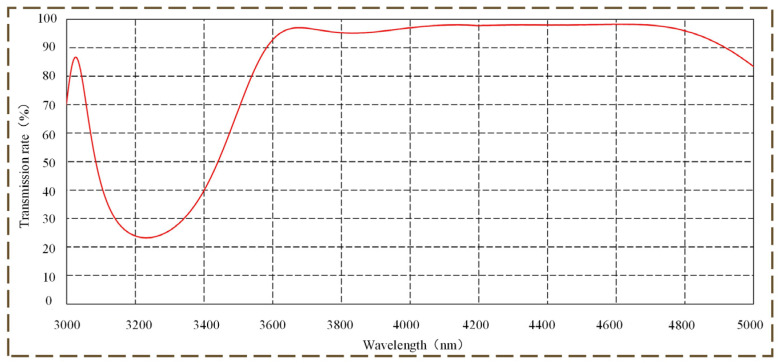
Measured transmittance of coated HMID.

We believe the disparity between HMID and nominal NETD values is mostly attributed to the following factors:

1. The blackbody emissivity does not reach the predicted 100%. When light of infrared wavelengths propagates through a reflecting parallel light tube with parameters ranging from 0.85 to 0.95 under laboratory circumstances, and energy loss occurs. Additionally, there is some energy loss in HMID manufactured on germanium substrates in the case of coating, Figure 11 depicts the observed transmission rate of HMID.
2. The thermal imaging system accepts the signal when the radiation difference between the target and the background corresponds to distinct target temperatures and varied background temperatures. System noise may rise when the detector is at a greater ambient temperature, hence the measured NETD value may be affected by the temperature of the environment.
3. The HMID has an F-number of 4.21, whereas the IR-cooled detector has an F-number of 4. According to the image plane illumination relationship of the optical system, it is stated as Equation (8):

(8) $$E_{image}=K\frac{\pi L}{{\left(F/\#\right)}^{2}}{\left(\frac{n^{’}}{n}\right)}^{2}$$

where *K* is the optical system’s passing coefficient, computed as the ratio of outgoing light energy to incident light energy. *L* is the luminosity, *F/#* is the system’s F-number, *n* is the refractive index of the air, and *n’* is the refractive index of the material.

Due to an increase in system F-number, the system image plane receives about 10% less energy, resulting in a disparity between the measured optical system NETD value and the detector.

In this section, we measure the NETD values of a single-lens imaging system utilizing HMID lenses and evaluate the causes for the disparity between the NETD and the measured and predicted values of the detector, which are mostly attributed to environmental factors. A more realistic model will be developed in the future. However, the NETD of our suggested single-lens imaging system is already similar to that of other uncooled IR detectors, proving that our proposed HMID lens design process is accurate.

## 5. Conclusions

In conclusion, the innovative achromatic method was used to an infrared optical system employing a combination of HODS and FODS to eliminate type 1 LCA in the mid-wave infrared band, hence providing good image performance at the design end, the LCA can be minimized by up to 30 times. We have manufactured this lens using the diamond turning method and conducted experimental validation, successfully validated the single-lens imaging function of HMID and determined that the difference between the measured NETD of the optical system and the detector is due to the machining error and the realistic scenario, but the NETD of the optical system remains sensitive. Design and experimental results demonstrated that the single-lens imaging approach employing HMID presented in this study is viable and effective. This method is applicable to imaging systems that employ the microstructured lenses discussed in this research. This study simply validates and assesses the single-lens imaging performance of HMID and its performance as an infrared system; as the experimental setup is established, field experiments will be carried out, the resulting images will be used to evaluate the imaging quality, and more accurate IR system models will be enhanced.

## Figures and Tables

**Table 1:** Parameters of the optical system.

Wavelength	Field of View	Radius	Focal Length	F/#	Material
3.7~4.7 (μm)	0.5°	34.876 mm	296.232 mm	4.21	Germanium

**Table 2:** Parameters of the HMID.

	Zone 1	Zone 2	Zone 3
**ZONAL RADIUS (MM)**	20.098	28.445	34.876
**SURFACE RADIUS (MM)**	915.028	915.815	916.675
**CONIC**	−3.000036	−3.000567	−3.000288
**A_FODS_2**	−52.857	−55.029	−83.971
**A_FODS_4**	7.441	7.255	215.162
**A_FODS_6**	−7.227	−2.613	−607.146
**A_FODS_8**	28.704	5.036	736.02
**A_FODS_10**	−39.673	−3.446	−313.766

Where Zonal radius is the size of each region’s radius and Surface radius is the region’s radius of curvature. A_FODS_2 is the second-term coefficient of FODS. A_HODS_4 is the fourth-term coefficient of HODS. A_FODS_6 is the sixth-term coefficient of FODS. A_FODS_8 is the eighth-term coefficient of FODS.

**Table 3:** Data of HMID sensitivity.

	Thicker Thickness	Thinner Thickness	Wider Width	Narrower Width
**ZONE 1**	0.462 μm	0.235 μm	10.68 μm	21 μm
**ZONE 2**	0.6396 μm	0.75 μm	24.33 μm	20.55 μm
**ZONE 3**	0.533 μm	1.55 μm	41 μm	13.99 μm

**Table 4:** NETD measurements of blackbody and HMID.

Temperature Differences (K)	0	0.5	1	1.5	2	2.5	3	3.5	4	4.5
**NETD of Blackbody (mK)**	16.3	16.4	16.5	16.6	16.6	16.8	16.9	17	17.1	17.2
**NETD of HMID (mK)**	51.8	51.2	53.1	51.7	51.6	51.6	52	52.2	54.8	54

## Data Availability

The data are available within the article.
